# Crystal structure and peculiarities of microwave parameters of Co_1−*x*_Ni_*x*_Fe_2_O_4_ nano spinel ferrites

**DOI:** 10.1039/d3ra04557a

**Published:** 2023-09-07

**Authors:** Marwa M. Hussein, Samia A. Saafan, H. F. Abosheiasha, Di Zhou, D. S. Klygach, M. G. Vakhitov, S. V. Trukhanov, A. V. Trukhanov, T. I. Zubar, K. A. Astapovich, Hesham M. H. Zakaly, Moustafa A. Darwish

**Affiliations:** a Physics Department, Faculty of Science, Tanta University Tanta 31527 Egypt; b Engineering Physics and Mathematics Department, Faculty of Engineering, Tanta University Tanta 31511 Egypt; c Electronic Materials Research Laboratory, Key Laboratory of the Ministry of Education & International Center for Dielectric Research, School of Electronic Science and Engineering, Xi'an Jiaotong University Xi'an 710049 China; d South Ural State University Chelyabinsk 454080 Russia; e Smart Sensor Laboratory, National University of Science and Technology MISIS 119049 Moscow 4, Leninsky ave. Russia trukhanov86@mail.ru; f SSPA “Scientific and Practical Materials Research Centre of NAS of Belarus” 220072 Minsk 19, P. Brovki str. Belarus ks-sd@mail.ru; g L.N. Gumilyov Eurasian National University Astana 010000 Kazakhstan; h Istinye University, Faculty of Engineering and Natural Sciences, Computer Engineering Department Istanbul 34396 Turkey; i Institute of Physics and Technology, Ural Federal University 620002 Yekaterinburg Russia

## Abstract

Nanosized spinel ferrites Co_1−*x*_Ni_*x*_Fe_2_O_4_ (where *x* = 0.0–1.0) or CNFO have been produced using a chemical method. The crystal structure's characteristics have been determined through the utilization of X-ray diffraction (XRD). It has been demonstrated that all samples have a single phase with cubic syngony (space group *Fd*3̄*m*). The lattice parameter and unit cell volume behavior correlate well with the average ionic radii of Co^2+^ and Ni^2+^ ions and their coordination numbers. Thus, an increase in the Ni^2+^ content from *x* = 0.0 to *x* = 1.0 leads to a decrease in the lattice parameter (from 8.3805 to 8.3316 Å) and unit cell volume (from 58.86 to 57.83 Å^3^). Elastic properties have been investigated using Fourier transform infrared (FTIR) analysis. The peculiarities of the microwave properties have been analyzed by the measured *S*-parameters in the range of 8–18 GHz. It was assumed that the energy losses due to reflection are a combination of electrical and magnetic losses due to polarization processes (dipole polarization) and magnetization reversal processes in the region of inter-resonant processes. A significant attenuation of the reflected wave energy (−10 … −21.8 dB) opens broad prospects for practical applications.

## Abstract 2

Nanosized spinel ferrites Co_1−*x*_Ni_*x*_Fe_2_O_4_ (where *x* = 0.0–1.0) or CNFO have been produced using a chemical method.

## Graphical abstract

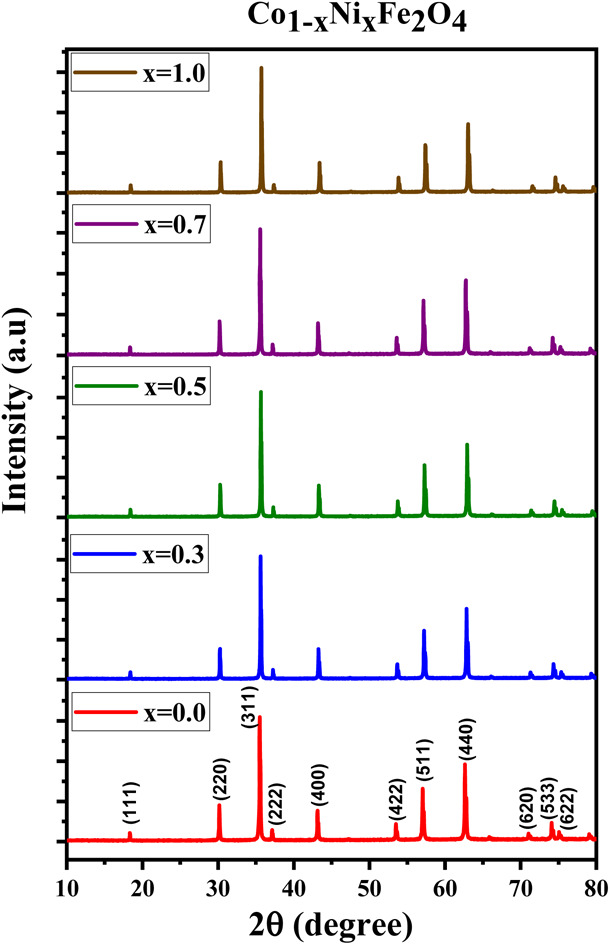

## Introduction

In recent years, multicomponent oxide systems have garnered significant practical and scientific importance, leading to increased attention from scientists.^1–5^ The notable interest of the scientific community can be associated with a significant correlation between the chemical composition of the oxide compound (cation ratio, anionic stoichiometry), local crystal structure, and its optical, electrical, microwave, and magnetic characteristics.^6–10^ The practical importance of such studies is due to the prospects for the wide use of multicomponent oxide systems in medicine, electronics, industry, ecology, *etc.* It should be clarified that ferrites, multicomponent oxide systems based on iron ions, are best studied in this class of compounds and most often find practical applications.^11–15^ Ferrites contain iron oxide (Fe_2_O_3_) as the main component, which endowes them with excellent magnetic properties. Depending on the formed crystal structure, there are four main groups of ferrites: spinels, garnets, orthoferrites, and hexaferrites.

The general formula for spinel ferrites is AB_2_O_4_, where A is a divalent ion (for example, Co^2+^, Ni^2+^, Cu^2+^, Mn^2+^, *etc.*) and B is a trivalent ion (usually Fe^3+^). Spinels are classified as soft magnetic materials due to their low coercivity and high magnetization properties.^16–18^ The distribution of cations between tetrahedral (A) and octahedral (B) positions has a significant impact on the crystal structure, microwave, and magnetic properties of spinel ferrites.^19,20^ This aspect makes it possible to use spinel ferrites in ecology and biomedicine, electronics, and industry for various purposes, including targeted drug delivery,^21^ MRI,^22^ battery cathode materials,^23^ longitudinal recording media,^24^ and anodes,^25^ gas sensors,^26^ antennas,^27^ microelectronics.^28^

Ferrites based on nickel ions have outstanding magnetic and electrical characteristics (low eddy current loss and coercivity, high magnetic permeability and mechanical hardness, extremely high electrical resistivity, and high operating frequency). Therefore, they are subjected to diversified research. The potential applications of nickel-based ferrites are numerous and promising, such as high-density and high-storage devices, transformers, microwave devices, magnetic fluids, *etc.* Due to outstanding magnetoresistive properties, nickel-cobalt ferrites occupy a special place in the spinel group. The cobalt-based ferrite has the formula CoFe_2_O_4_, exhibits the highest magnetocrystalline anisotropy among all spinels, and is therefore characterized as a hard-magnetic material.^29,30^ At the same time, nickel-based ferrite with the formula NiFe_2_O_4_ has a low coercive force and high saturation magnetization, which makes it a soft magnetic material.^31^ Both cobalt and nickel ferrites crystallize into inverse spinels when trivalent iron cations equally occupy both tetrahedral and octahedral positions. It is possible to combine the properties of soft and hard ferrites, for example, as occurs in nickel-cobalt ferrite (Co_1−*x*_Ni_*x*_Fe_2_O_4_ or CNFO), to expand the possibilities of using spinels in high-frequency devices and data storage technologies.^32–34^ Many studies^35–38^ show how the crystal structure, morphology, microwave, electrical and magnetic characteristics of CNFO change depending on the conditions and method of synthesis and the ratio of Co^2+^/Ni^2+^.

It is worth mentioning that the incorporation of Co and Ni into ferrite structures, specifically in the form of ferrites Co_1−*x*_Ni_*x*_Fe_2_O_4_ spinel ferrites, has been a subject of interest due to their distinct magnetic properties. The rationale behind introducing these elements can be elucidated as follows: (1) magnetic anisotropy: cobalt ions (Co^2+^) are known for imparting high magnetic anisotropy to ferrites. This anisotropy is pivotal for enhancing electromagnetic wave absorption, particularly in the microwave frequency range. (2) Tuning magnetic properties: the substitution of Co by Ni allows for systematic tuning of the magnetic properties of the ferrite. Nickel ions (Ni^2+^) typically have a smaller magnetic moment than cobalt ions. By varying the ratio of Co to Ni, one can achieve a broad range of magnetic properties suitable for different applications. (3) Enhancing saturation magnetization: cobalt ferrites generally possess high saturation magnetization. By blending Co with Ni in the ferrite structure, it's possible to maintain a high level of saturation magnetization, which is beneficial for electromagnetic wave absorption. So, incorporating both Co and Ni into the ferrite structure thus provides a balanced set of properties, achieving enhanced magnetic anisotropy and tunability, making the material more versatile for electromagnetic wave absorption applications.^29–38^

The authors of this study discuss the crystal structure and peculiarities of microwave parameters of Co_1−*x*_Ni_*x*_Fe_2_O_4_ (where *x* = 0.0–1.0) nano-spinel ferrites or CNFO synthesized by the citrate-nitrate auto-combustion method.

## Experimental

### Synthesis

The following steps have been used to synthesize CNFO nanoparticles using the citrate-nitrate auto combustion approach: initially, a combination of chemical reagents including Co(NO_3_)_2_·6H_2_O (≥98.5% purity, sourced from Qualikems), Ni(NO_3_)_2_·6H_2_O (≥98% purity, sourced from Advent Chembio Pvt. Ltd), Fe(NO_3_)_3_·9H_2_O (≥98.5% purity, sourced from Qualikems), and C_6_H_8_O_7_ (≥99.5% purity, sourced from Oxford Lab Fine Chem LLP) have been adjusted in weights, such that the ratios of the used anhydrous citric acid to trivalent metal ions to and divalent metal ions were 3 : 2 : 1.^39–41^ These nitrate salts were dissolved in distilled water and stirred for 15 minutes without heating to create a homogeneous mixture before adding the citric acid. Finally, ammonium hydroxide was added to the mix in drops while the stirring continued until the pH reached approximately 7.0.^42,43^ After removing the magnet, the mixture was heated to about 120 °C for three hours. A formed viscous gel has been self-ignited to give a fine and brown powder of the ferrite eventually. The material has been grained by agate mortar, then pressed to discs and sintered at 900 °C for 4 hours. Finally, the disc-shaped CNFO samples were ground again and prepared for characterization. The following formula can characterize the synthesis of the studied CNFO compositions:(1−*x*)Co(NO_3_)_2_·6H_2_O + (*x*)Ni(NO_3_)_2_·6H_2_O + 2Fe(NO_3_)_3_·9H_2_O + 3C_6_H_8_O_7_ → Co_1−*x*_Ni_*x*_Fe_2_O_4_ + emitted gasses↑

**Scheme 1:**
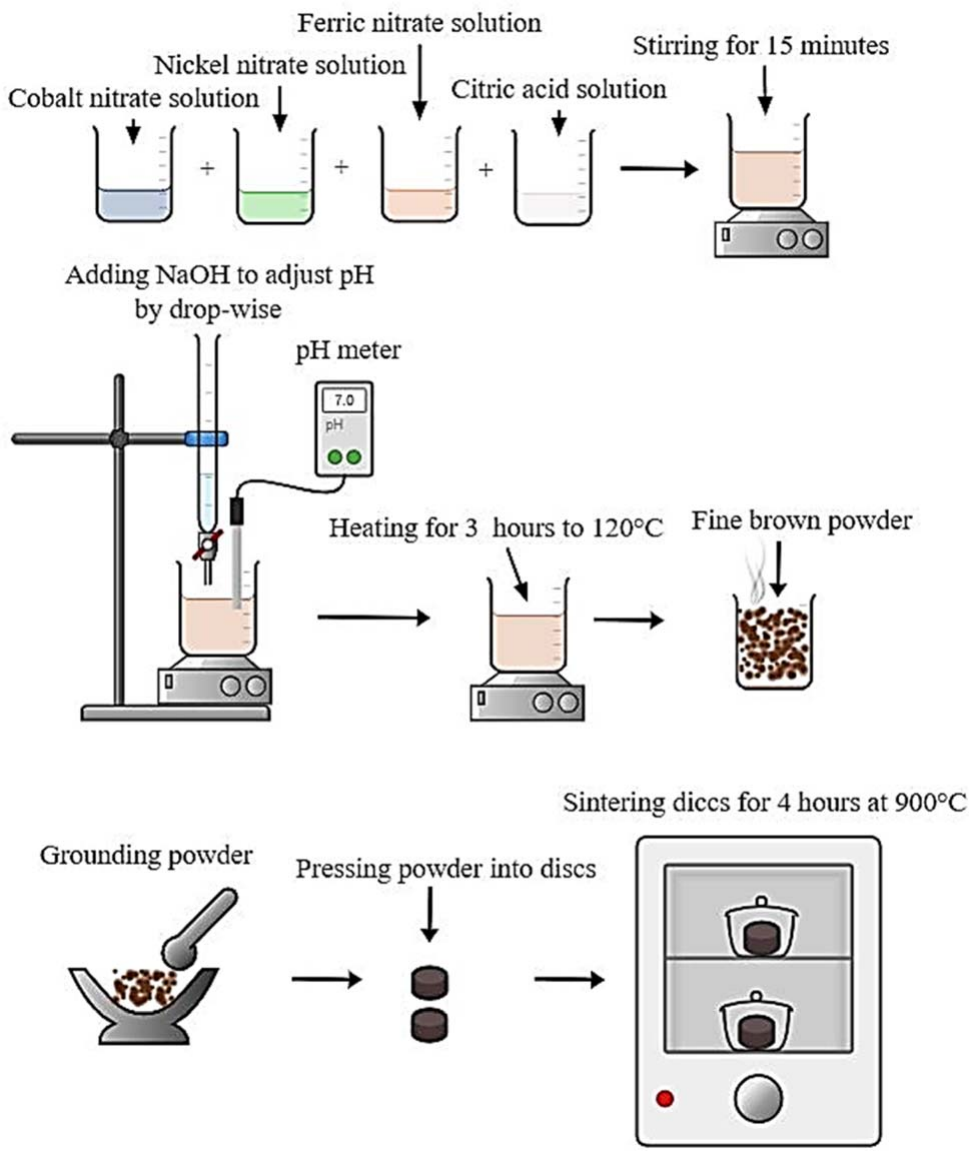
Synthesis of CNFO (*x* = 0.0–1.0).

Scheme 1 shows the preparation method of CNFO nano ferrites.

### Characterization

CNFO samples were studied using various analytical methods, such as XRD PANALYTICAL co. Xpert Pro system in the Central Metallurgical Research and Development Institute in Helwan with a Cu-K_α_ target and a wavelength of 1.54 Å, 54 kV, and 40 mA. FTIR was performed *via* FTIR spectroscopy (Bruker Tensor 27) in the range of 200–5000 cm^−1^ in the Scientific Research Center and Measurements of Tanta University. CNFO microstructure was investigated with SEM (Zeiss EVO 10, Oberkochen, Germany). The chemical composition of nanosized CNFO was studied using EDXS (AZtecLive Advanced Ultim Max 40 detector, Oxford Instruments, Bognor Regis, UK). The average particle size of CoFe_2_O_4_ was estimated by TEM (JEM-2100 instrument at the National Research Center in Cairo). The vector network analyzer (Agilent) has investigated the microwave properties of ferrites. The reflection loss was calculated with the methodology and equations sourced from referenced literature.^44–47^

## Results and discussion

### Crystal structure and elastic properties

**Fig. 1:**
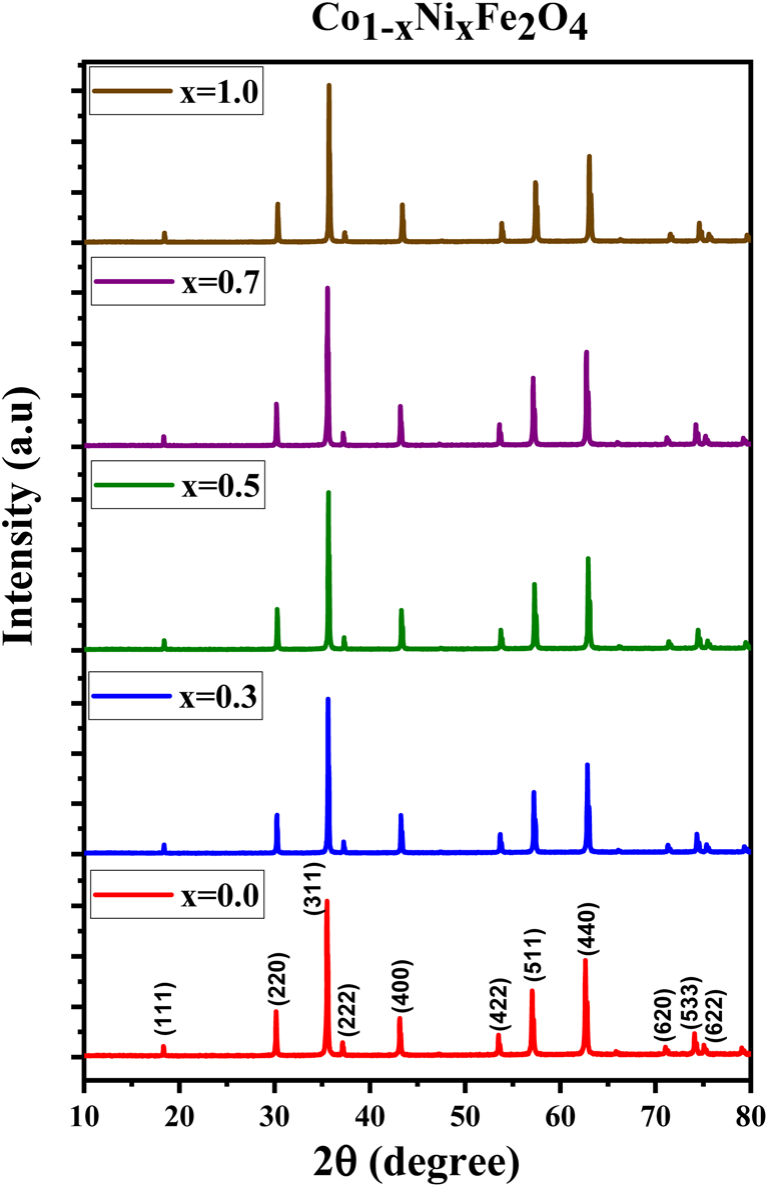
Spectra of the X-ray diffraction of CNFO (*x* = 0.0–1.0).

Fig. 1 displays the X-ray diffraction (XRD) patterns of the synthesized CNFO. The CoFe_2_O_4_ sample (CNFO composition with *x* = 0.0) has the most intense diffraction peaks located at 2*θ* = 18.33°, 30.14°, 35.49°, 37.13°, 43.13°, 53.51°, 57.03°, 62.36°, 71.04°, 74.09°, and 75.09° corresponding to the (111), (220), (311), (400), (422), (511), (440), (620), (533), (002), and (622) planes respectively. These data agree with the CoFe_2_O_4_ standard card (JCPDS no. 01-086-8870), which confirms the successful synthesis of spinel ferrite with cubic crystal structure without any detectable impurities. The planes listed above are also established for the rest of the CNFO samples, which demonstrates the successful synthesis of spinel ferrites with cubic crystal structure without any interstitial phases according to the standard card of Ni_0.5_Co_0.5_Fe_2_O_4_ (JCPDS no. 01-083-6066) and (JCPDS no. 00-066-0246), NiFe_2_O_4_ (JCPDS no. 01-076-6120).

These results benefit the selected synthesis and grafting processes, which are expected to achieve better material performance for intended applications. The crystallinity of CNFO composites is also confirmed by the fact that the detected XRD peaks are sharp and narrow. Moreover, the main characteristic peaks of the CNFO compounds undergo a slight shift towards larger 2*θ* angles with an increase in the Ni^2+^ concentration. Such a shift can be associated with a change in *d*-spacing, determined by the lattice parameter and volume, and is explained by the fact that the nickel ion has smaller ionic radii (0.63 Å) with regard to the cobalt ion (0.74 Å).^48^ This also confirms that Ni^2+^ is a good substitute for Co^2+^ in CNFO. The crystal size was determined using the Scherrer formula:
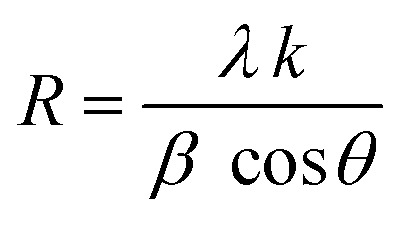
where *λ* is the X-ray wavelength (= 1.54 Å), *k* is a constant, and its value depends on the crystallite shape (= 0.89 for cubic crystals), *β* denotes the FWHM (full width at half maximum) of the main peak representing the planes (311), *θ* represents the Bragg angle.
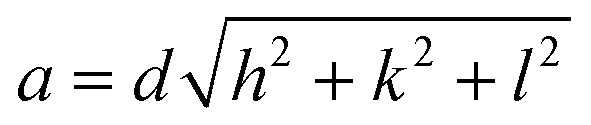
where *d* is the interplanar spacing, and it can be determined using Bragg's equation as follows:
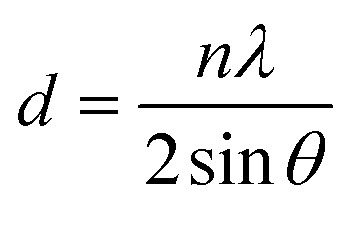


The following equation was used to determine the X-ray density for CNFO samples (*D*_*x*_):
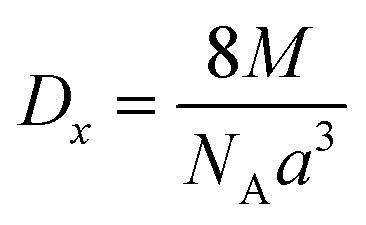
where *M* represents the molecular weight of the sample, *N*_A_ is Avogadro's number, and *a*^3^ represents the unit cell volume.

Furthermore, jump length (the distance between magnetic ions) in the tetrahedral A-site *L*_A–A_ (Å), octahedral B-site *L*_B–B_ (Å), and shared sites *L*_A–B_ (Å) have been estimated using the following equations:^39,49^
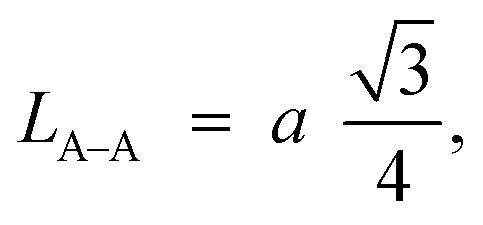

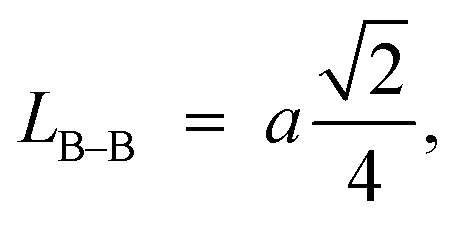

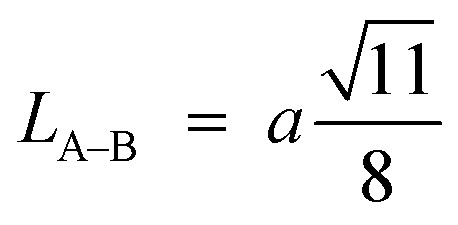


The estimation of dislocation density, defined as the number of dislocation lines per unit volume of crystal, represented by the symbol *δ*, can be achieved through the utilization of the following equation:
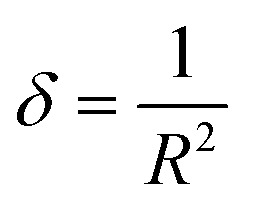


Lattice strain (*ε*) caused by crystal distortions and defects in the obtained CNFO ferrites was calculated by the formula:^50,51^
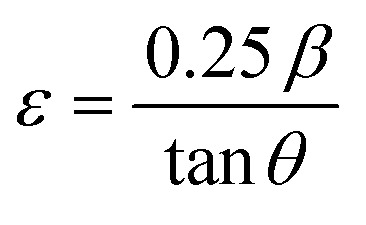


The estimation of the mean ionic radius per molecule for the tetrahedral and octahedral sites, denoted as *r*_A_ and *r*_B,_ respectively, has been calculated by utilizing the cation distribution for each composition and using the following relations:*r*_A_ = [*f*(Co^2+^)·*r*(Co^2+^) + *f*(Ni^2+^)·*r*(Ni^2+^) + *f*(Fe^3+^)·*r*(Fe^3+^)]*r*_B_ = 0.5[*f*(Co^2+^)·*r*(Co^2+^) + *f*(Ni^2+^)·*r*(Ni^2+^) + *f*(Fe^3+^)·*r*(Fe^3+^)]where *f* represents the fractional concentration, *r* refers to the ionic radius of the respective cation on the respective site.

The theoretical value of the lattice parameter a_Th_ was calculated for all CNFO compositions from the following formula:^52,53^*a*_Th_ = 1.5396[(*r*_A_ + *r*_O_) + 1.732(*r*_B_ + *r*_O_)]where *r*_O_ is the oxygen ion radius.

**Fig. 2:**
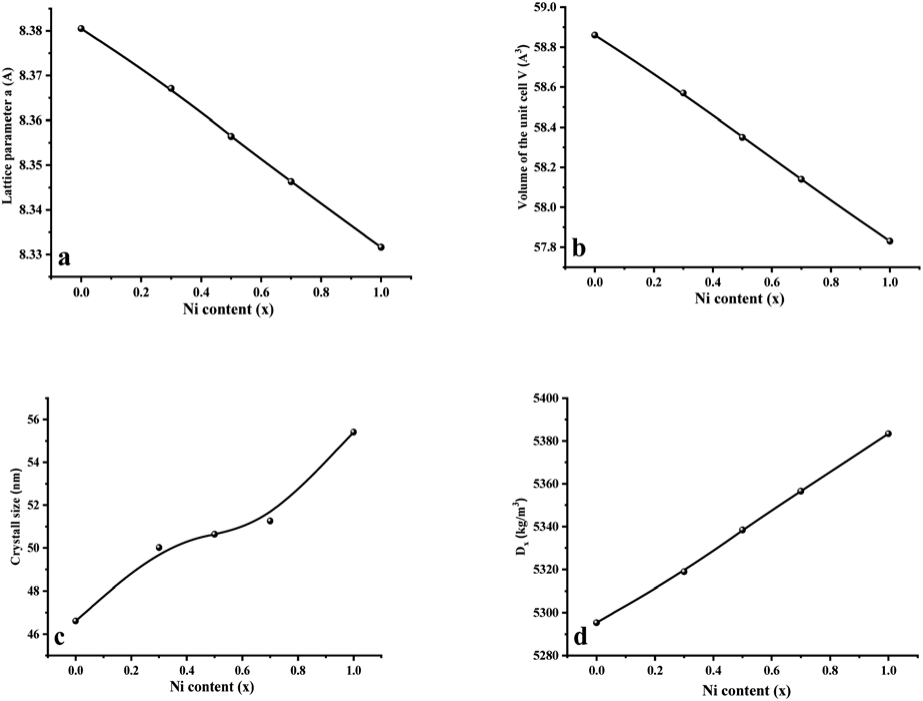
Main structural parameters of CNFO (*x* = 0.0–1.0) obtained from XRD data. (a) Lattice parameter *a*. (b) Volume of the unit cell *V*. (c) Average crystal size. (d) X-Ray density.

Fig. 2 displays the concentration dependencies of the main structural parameters of CNFO (*x* = 0.0–1.0) obtained from the X-ray diffraction (XRD) data. Some parameters are listed in Table 1. The crystal size varies from 46.61 to 55.41 nm, as demonstrated in Fig. 2c. A monotonic increase is observed with an increase in Ni^2+^ content, which is expected at a sintering temperature of 900 °C for 4 hours. This can be explained by decreasing lattice strain, which usually contributes to increased crystallite size.^53^

**Table 1:** Structural parameters of the CNFO: ionic radius of tetrahedral – *r*_A_ (Å) and octahedral sites – *r*_B_ (Å), theoretical lattice constant – *a*_Th_ (Å); interlattice and intralattice distance – *L*_A–A_ (Å), *L*_B–B_ (Å), *L*_A–B_ (Å), dislocation density – *δ* (m^−2^), lattice strain – *ε* (lin^−2^ m^−4^)

*x*	*r*_A_ (Å)	*r*_B_ (Å)	*a*_Th_ (Å)	*L*_A–A_ (Å)	*L*_B–B_ (Å)	*L*_A–B_ (Å)	*δ* (m^−2^)	*ε* (lin^−2^ m^−4^)
0.0	0.4904	0.644	8.442	3.628	2.962	3.474	4.60106 × 10^14^	0.002412
0.3	0.4910	0.636	8.421	3.623	2.958	3.468	3.99576 × 10^14^	0.002241
0.5	0.4903	0.632	8.409	3.618	2.954	3.464	3.89872 × 10^14^	0.002212
0.7	0.4902	0.627	8.395	3.614	2.951	3.461	3.80557 × 10^14^	0.002190
1.0	0.4901	0.619	8.376	3.607	2.945	3.454	3.25657 × 10^14^	0.002017

As the nickel content increases, a bigger Co^2+^ ion (0.745 Å at the octahedral site and 0.580 Å at the tetrahedral site) is substituted by a smaller Ni^2+^ ion (0.690 Å at the octahedral site and 0.550 Å at tetrahedral site) in the CNFO ferrites^54^ Thus, there is a linear decrease in the lattice parameter and unit cell volume, with increasing Ni^2+^ concentration, following the trend expected from Vegard's rule.^55^ The calculated values of theoretical lattice parameters correlate well with the established (*a*_Th_) and (*a*) values, which confirms the reliability of the proposed distribution of cations for the CNFO system. A small difference between the calculated lattice parameters (*a*_Th_) and their experimental values (*a*) was expected due to the changes in the distribution of cations among all ions.^56^ Both cobalt and nickel ferrites crystallize into inverse spinels when Co^2+^ and Ni^2+^ cations prefer to occupy octahedral (B) positions.

In contrast, the Fe^3+^ cations exhibit distribution across both octahedral (B) and tetrahedral (A) positions. This distribution can be influenced by many factors (*e.g.*, annealing temperature), which can cause a small amount of Co^2+^ and Ni^2+^ cations to move to (A) positions. Also, it is observed that *r*_A_ and *r*_B_ values show almost a decreasing trend with an increase in nickel concentration. These changes in *r*_A_ and *r*_B_ are associated with different distributions of cations in tetrahedral and octahedral positions. This means that the concentration of Co^2+^ (Ni^2+^) cations in the A- and B-positions increases (reduces) the cation redistribution and the migration of some of the Fe^3+^ ions from the A- positions to the B-positions. Since cations' distribution strongly influences ferrites' magnetic properties, the assumption made is also investigated, considering the obtained magnetic characteristics, which will be presented later in this study.

The observed reduction in the unit cell volume (lattice parameter (*a*)) exceeds the reduction in molecular weight. The X-ray density (*D*_*x*_) increases with an increase in the nickel concentration since the atomic mass of Co^2+^ (58.933 AMU) is very close to that of Ni^2+^ (58.693 AMU). As a result of the formation of pores during disc-shaped pressing of CNFO samples and sintering processes at high temperatures, X-ray density values (*D*_*x*_) are observed to be higher than the measured density values (*D*). The porosity of the CNFO system is low and tends to increase with increasing nickel concentration. This can be attributed to abnormal compaction and imperfection of the crystal structure, which depends on stoichiometry, synthesis method, heat treatment conditions, and the interplay between *D* and *D*_*x*_.^53^ As the concentration of Ni^2+^ ions increases, the number of atomic defects, such as dislocation density (*δ*) and strain (*ε*), are observed to decrease, displaying an inverse correlation with the crystallite dimensions. This phenomenon aligns with the expected patterns. The contraction of lattice parameters is driven by a decrease in the proximity between magnetic ions, a process referenced in previous literature.^56^ This spatial shift results from the cation redistribution that occurs due to the substitution of Ni ions.^53^

Consequently, *L*_A–A_, *L*_B–B_, and *L*_A–B_ values diminish with a growing concentration of nickel, which is consistent with theoretical predictions based on relevant equations. Observations reveal a clear pattern: the value of *L*_A–A_ consistently surpasses *L*_B–B_. This suggests that the likelihood of electron transition between ions residing at A and B sites is lower than between ions located at the same B site.^55,56^

**Fig. 3:**
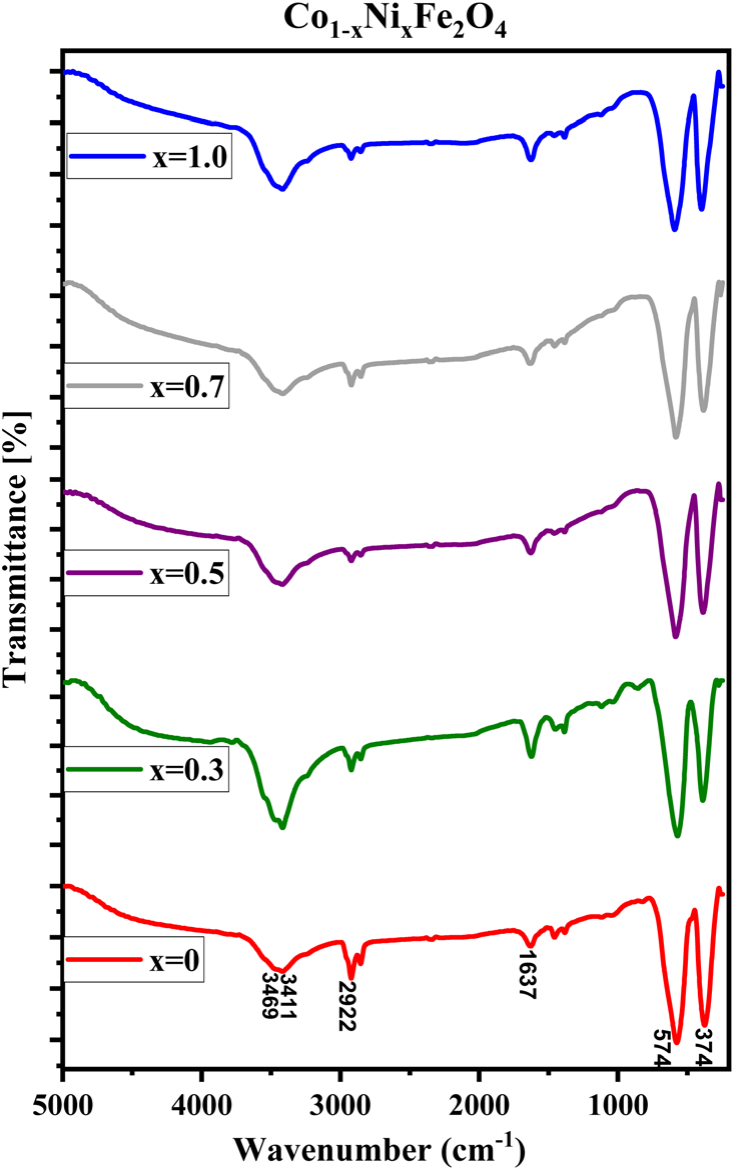
FTIR spectra of CNFO (*x* = 0.0–1.0).

FTIR spectra of CNFO (*x* = 0.0–1.0) samples are illustrated in Fig. 3. The vibrational bands located around 575–595 cm^−1^ and 375–395 cm^−1^ are attributed to the stretching vibrations of the tetrahedral and octahedral groups, respectively. These vibrational modes are commonly referred to as (*ν*_1_) and (*ν*_2_). The presence of these two prominent metal–oxygen absorption bands is a fundamental characteristic observed in all FTIR spectra of spinel ferrite nanoparticles documented in the literature.^55,57,58^ The bands observed at 1637 and 2922 cm^−1^ indicate the presence of additional O–H (or C–H/C–C) groups, confirming the existence of interlayer water and the oscillations of H–O–H bonds. The stretching mode of O–H bending vibration is attributed to the absorption band observed at 3411 cm^−1^.

The identifiable band suggests the formation of hydrogen bonds amongst hydroxyl groups, providing evidence for the presence of either free or adsorbed water within the sample. A broad absorption band detected at 3469 cm^−1^ is ascribed to the stretching vibrations of the O–H bond in water molecules present within the interstitial spaces of the layers.^42,57^ Minute changes in the intensity and minor spectral shifts in the two primary characteristic absorption bands of the ferrites are noted, as presented in Table 2 and Fig. 3. The reason for these observed anomalies may be correlated with factors such as changes in the effective atomic mass, bond lengths, force constants, and the electronegativity of the cations.^59^ The shift observed in the position of the absorption band, originating from the tetrahedral and octahedral sites, is linked to the force constant. This constant is directly proportional to the atomic number of the metal ions, the atomic number of the oxygen ions, and the length of the metal–oxygen bond, respectively. The force constants for the tetrahedral site, denoted as *F*_1_, and the octahedral site, denoted as *F*_2_, can be computed using the provided equations:*F*_1_ = 4π^2^*c*^2^*ν*_1_^2^*μ**F*_2_ = 4π^2^*c*^2^*ν*_2_^2^*μ*where *c* represents the velocity of light in vacuum, while *μ* represents the reduced mass of the system comprising oxygen and metal, which is equivalent to 2.061 × 10^−23^ g.

**Table 2:** FTIR absorption band values, force constants *F*_*ν*1_ (dyne per cm) and *F*_*ν*2_ (dyne per cm) at A- and B-sites, average force constant *F*_av_ (dyne per cm), elastic stiffness constants *C*_11_ (GPa) and *C*_12_ (GPa), longitudinal *V*_l_ (m s^−1^) and transverse *V*_s_ (m s^−1^) wave velocities, bulk modulus *B* (GPa), rigidity modulus *G* (GPa), Young modulus *E* (GPa), Poisson's ratio (*σ*), and Debye temperature *Θ*_D_ (K) of CNFO (*x* = 0.0–1.0)

*x*	*ν*_1_ cm^−1^	*ν*_2_ cm^−1^	*F*_*ν*1_ (dyne per cm)	*F*_*ν*2_ (dyne per cm)	*F*_av_ (dyne per cm)	*C*_11_ (GPa)	*C*_12_ (GPa)	*V*_l_ (10^3^ m s^−1^)	*V*_s_ (10^3^ m s^−1^)	*B* (GPa)	*G* (GPa)	*E* (GPa)	*σ*	*Θ*_D_ (K)
0.0	572.77	375.27	239 877.5	102 971.4	171 424.5	204.55	40.61	6.215	3.588	95.25	68.18	165.14	0.165	682.7
0.3	574.77	383.81	241 555.7	107 711.4	174 633.5	208.71	39.52	6.264	3.616	95.91	69.57	168.07	0.159	690.3
0.5	576.28	385.75	242 826.5	108 803	175 814.8	210.39	38.93	6.275	3.623	96.08	70.13	169.22	0.156	692.8
0.7	586.34	387.68	251 378.5	109 894.5	180 636.5	216.42	39.18	6.356	3.669	98.26	72.14	173.87	0.153	701.5
1.0	591.43	396.96	255 761.8	115 218.6	185 490.2	222.63	38.68	6.431	3.712	100	74.21	178.48	0.148	711.8

Table 2 consolidates the Fourier Transform Infrared (FTIR) data accrued from the analyzed samples. It is observed that the force constant values for *F*_1_ surpass those for *F*_2_, a phenomenon that aligns with expectations. This discrepancy can be attributed to variations in the stretching of bands between *ν*_1_ and *ν*_2_, intensified cationic interactions within the tetrahedral site, decreased interatomic distances, and the increased energy prerequisite for bond disruption.^60^

The modifications in the force constants *F*_1_ and *F*_2_ can be linked to the cation redistribution within the tetrahedral and octahedral sites, which occurs in correlation with variations in grain sizes.^55^

Their elastic characteristics indicate the isotropy and homogeneity of materials. Together, XRD and IR spectral analyses make their estimation easier. The elastic stiffness constants of the spinel ferrite system can be calculated using the following equations:
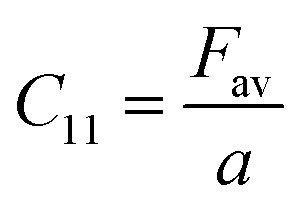

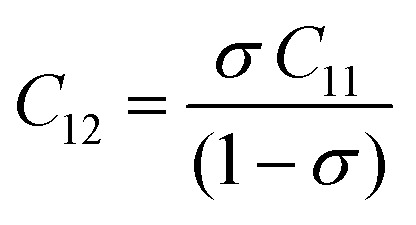
where *F*_av_ represents the average force constant of *F*_A_ and *F*_B_ while *σ* denotes the Poisson's ratio, which is dependent on porosity (*P*) given by:*σ* = 0.324 × (1–1.043*P*)

For solids exhibiting a cubic structure, the estimation of their elastic moduli – encompassing the bulk modulus (*B*), Young's modulus (*E*), and the shear modulus, also known as rigidity modulus (*G*) – can be conducted *via* the utilization of the computed stiffness constants. The corresponding equations used for these calculations are as follows:
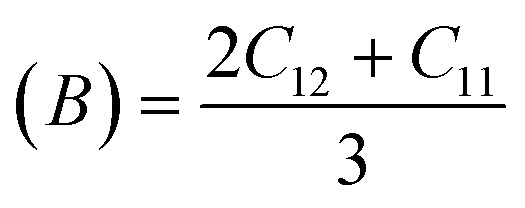

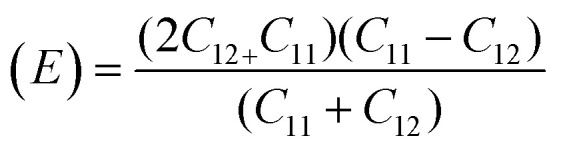

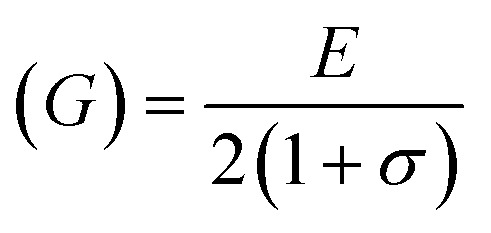


Moreover, the X-ray density (*D*_*x*_) and the stiffness constant *C*_11_ can be deployed to calculate the velocities of longitudinal and transverse elastic waves, represented by *V*_l_ and *V*_s_, respectively.

The calculations are guided by the subsequent equations, as indicated in the ref. 49 and 60:
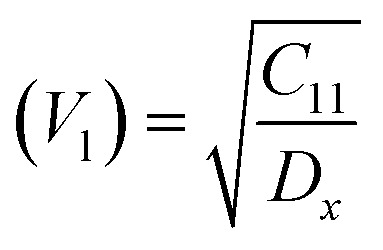

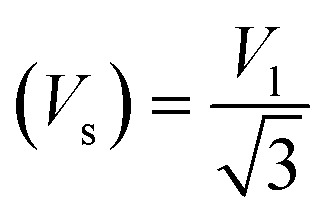


Finally, the Debye temperature *Θ*_D_ can be calculated using the formula:^61^
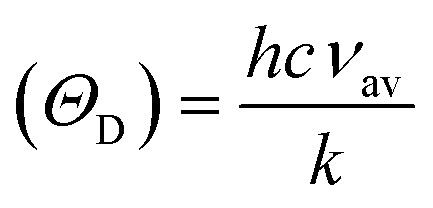
where *h* represents Plank's constant, *k* represents Boltzmann's constant, *c* is the speed of light, *ν*_av_ is the average value of wavenumbers for *ν*_1_ and *ν*_2_ at A and B-sites.

Table 2 presents all estimated values obtained from the calculations. The observed pattern of the stiffness constant *C*_11_ escalating with increasing Ni content can be interpreted as a consequence of the fortification of interatomic bonds amongst distinct atoms within the spinel lattice. Concurrently, the stiffness constant *C*_12_ displays a decrease, indicating a probable decline in the crystallinity of the samples with escalating Ni content. Both the longitudinal (*V*_l_) and transverse (*V*_s_) elastic wave velocities demonstrate an increase correlating with the rise in Ni content, a phenomenon attributable to an elevation in the average force constant. It is projected that *V*_l_ values will surpass *V*_s_ values due to the understanding that transverse waves necessitate lesser energy to instigate particle vibration perpendicular to the direction of wave propagation, in contrast to longitudinal waves that demand higher energy to stimulate particle vibration in the parallel direction.^61,62^ Poisson's ratio decreases as expected since the porosity values increase, as shown above in Table 2. It is well known that X-ray density affects elastic moduli.^63^ Therefore, since the values of X-ray density increase with increasing nickel content, as shown in Table 2, the elastic moduli *B*, *G*, and *E* increase as functions of density.

It has been observed that the Debye temperature shows an upward trend with increasing the concentration of nickel. The observed trend in the Debye temperature can be attributed to the rise in the wave number of vibrational bands of the infrared spectra, as well as a rise in the stiffness of the samples.^61^

**Fig. 4:**
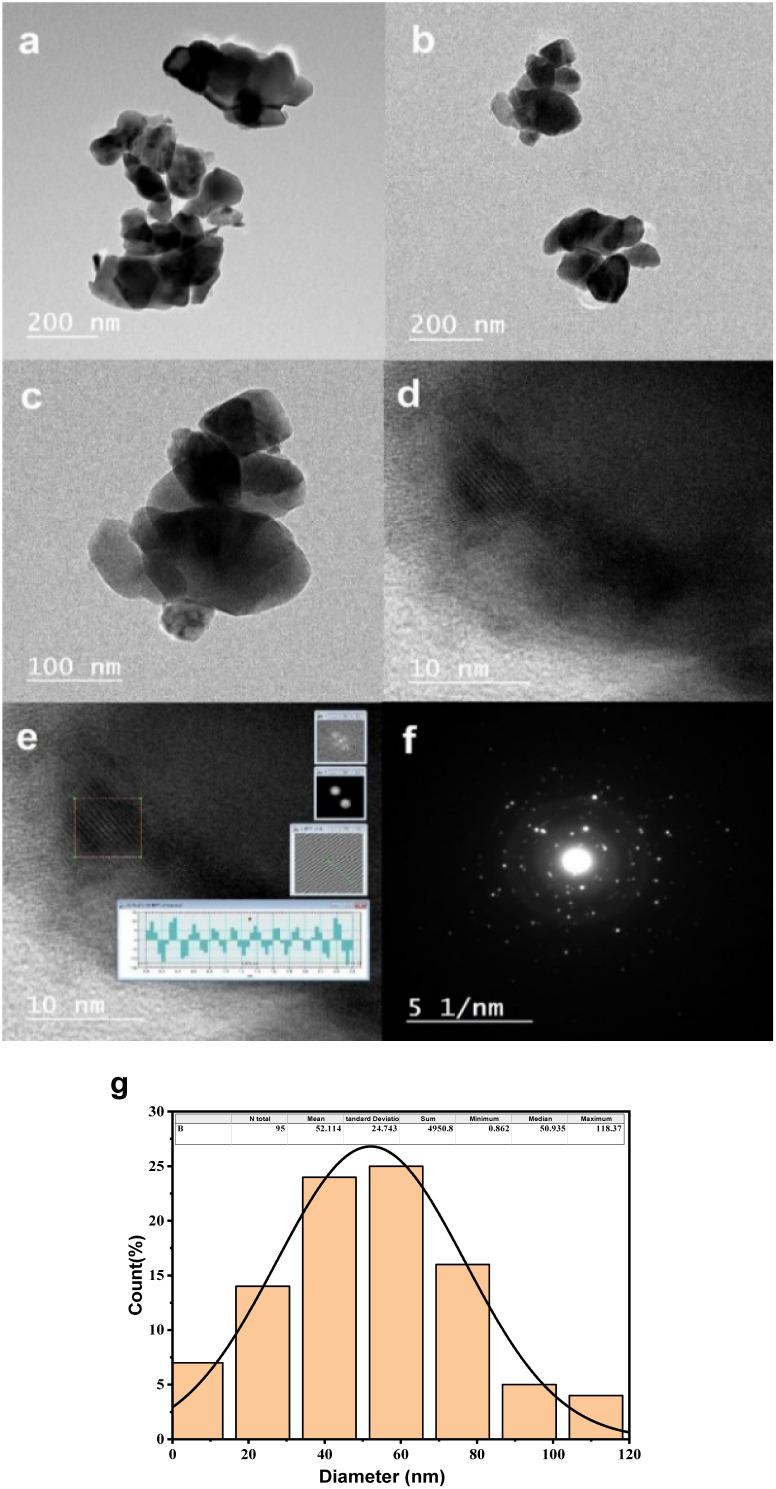
Electron microscopy data for CNFO (*x* = 0.0–1.0). (a–c) TEM micrographs of CNFO (*x* = 0.0) in the diffraction mode, (d and e) HRTEM images, (f) selected area electron diffraction (SAED) pattern, (g) histogram of particle size distribution.

Fig. 4(a)–(c) present Transmission Electron Microscopy (TEM) micrographs of CoFe_2_O_4_ captured in diffraction mode, while Fig. 4(d) and (e) depict High-Resolution Transmission Electron Microscopy (HRTEM) images. Fig. 4(f) demonstrates the Selected Area Electron Diffraction (SAED) pattern, and Fig. 4(g) illustrates a histogram representing the distribution of particle sizes.

The TEM micrographs reveal the existence of nano-scale particles and numerous agglomerations, consequences of magnetic inter-particle interactions. The identified interplanar spacing for CoFe_2_O_4_ is 0.2604 nm, corresponding to the (311) crystal plane's interplanar spacing. The histogram revealing the average particle size distribution is derived from the TEM images, facilitated by ImageJ software, and involves the analysis of a total of 95 particles within the TEM image. The mean particle size is identified as 52.1 nm, which aligns with the average crystallite size determined through the Scherrer equation, leveraging X-ray Diffraction (XRD) data. The minor disparity between the average sizes determined *via* XRD and TEM arises from the fact that XRD measures crystallite size. At the same time, TEM gauges the dimensions of whole particles. The SAED pattern reveals ring patterns, underscoring the crystalline nature of cobalt ferrite.

**Fig. 5:**
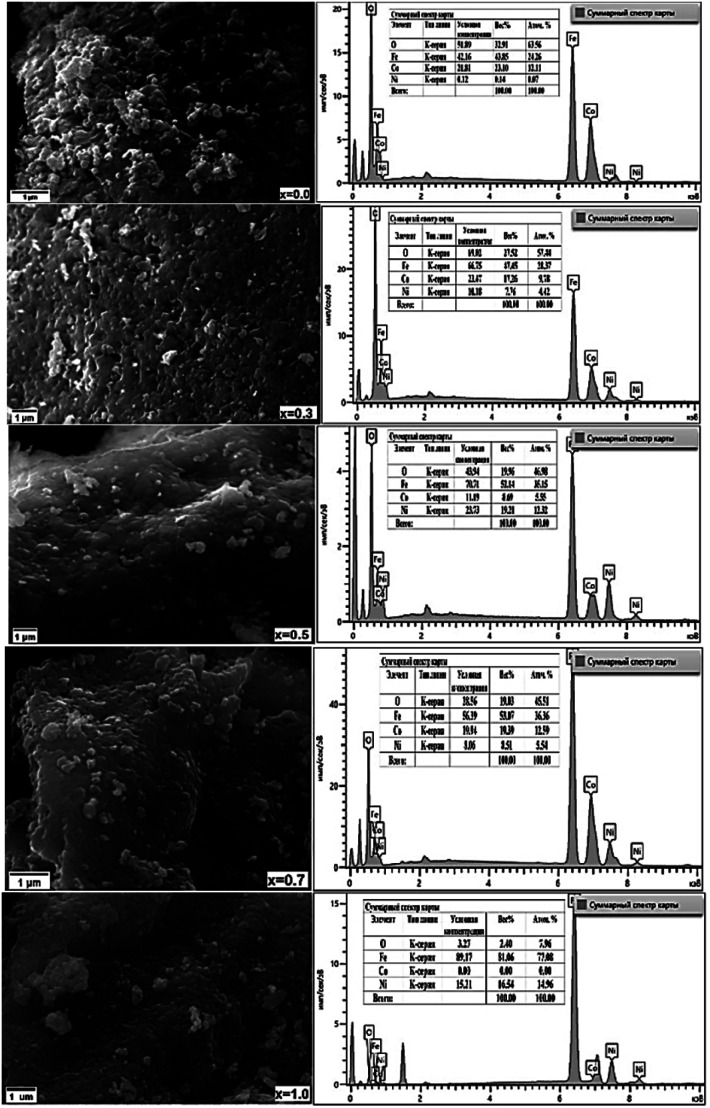
SEM micrographs (left column) and corresponding EDXS micrographs (right column) of CNFO (*x* = 0.0–1.0).

The surface topography and morphology of CNFO (*x* = 0.0–1.0) samples were investigated utilizing Scanning Electron Microscopy (SEM), with results illustrated in Fig. 5.

Analysis of the SEM imagery confirms that the samples possess a dense and homogenous structure, featuring fine spherical particles with irregularly oriented grain aggregations. The compositional analysis of the samples was carried out through Energy Dispersive X-ray Spectroscopy (EDXS). As demonstrated in Fig. 4, the findings verify the existence of Co, Ni, Fe, and O, affirming the successful, uncontaminated synthesis of the samples, devoid of any unintended elements. Furthermore, the observed escalation in the intensity of nickel depicted in the graphs, corresponding with the rise in Ni content, implies its successful integration within the CoFe_2_O_4_ ferrite structure.

### Microwave parameters

**Fig. 6:**
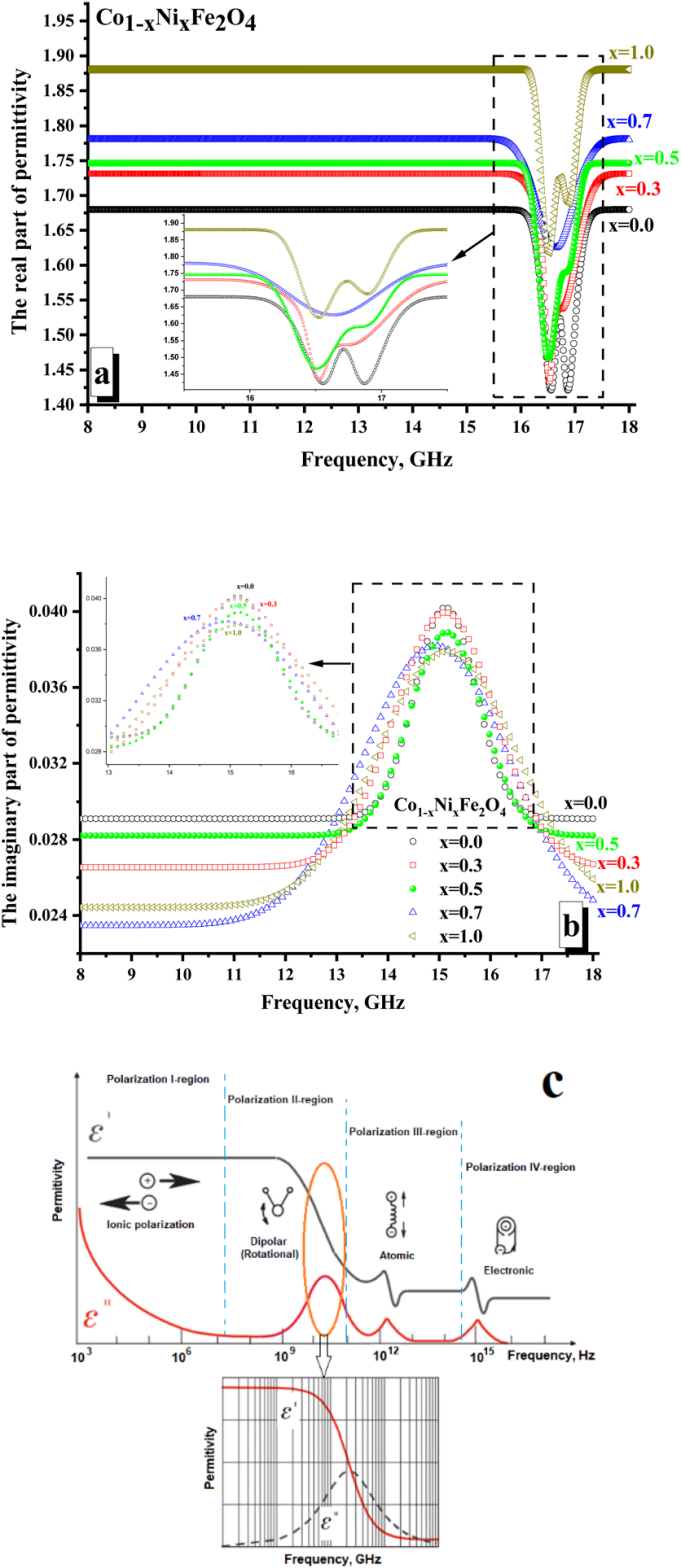
Frequency dependences of the permittivity of CNFO (*x* = 0.0–1.0). (a) Real part of the permittivity; (b) imaginary part of permittivity; (c) mechanism of the electrical losses in condensed matter in the high-frequency range.

Fig. 6 illustrates the frequency-dependent behavior of the permittivity for CNFO with (*x* = 0.0–1.0). The measurements were conducted within the frequency range of 8 to 18 gigahertz.

The values of the real and imaginary parts of the electric permittivity and magnetic permeability were calculated from the measured *S*-parameters. In Fig. 6a, one can analyze the behavior of the real part of the permittivity. It is shown that the *x* = 0.0 sample is characterized by the minimum values of the real part permittivity (∼1.65–1.68). It is noted that with increasing Ni^2+^ concentration, the values of permeability increase monotonically and are: ∼1.71–1.73 (for *x* = 0.3); ∼1.74–1.75 (for *x* = 0.5); ∼1.77–1.78 (for *x* = 0.7). The sample *x* = 1.0 (∼1.88–1.89) was characterized by the maximum value of the real part permittivity. It is shown that in the region of 16.3–17.5 GHz, local minima are noted for all the samples. For both samples with *x* = 0.0 and 1.0, two clear minima are noted. While for the rest of the samples, there is blurring by local minima (often with the formation of a wide plateau, as for *x* = 0.5). The amplitude of these minima correlates well with the nickel concentration. So, it can be noticed that the minimum value of the amplitude is typical for *x* = 0.0 (∼1.42), while the maximum value is noted for *x* = 1.0 (∼1.62).

The presence of local minima may be due to polarization losses (Fig. 6c). Thus, a significant reduction in the real part of the permittivity with an increase in the imaginary part (Fig. 6b demonstrates the presence of a peak in the frequency dependences of the imaginary part of the permittivity) may correspond to dipolar polarization. The polarization processes are attributed to the real part of the permittivity.

In contrast, the imaginary part is responsible for absorption due to electrical losses in the material. Relatively low values of the imaginary part of the permittivity indicate the absence of significant absorption due to electrical losses. This behavior of the electrical permittivity can be observed due to a change in the electronic configuration of the A-cation (substitution of a Co^2+^ ion by Ni^2+^ ion). Thus, the Ni^2+^ ion has the configuration of the outer electron shells 3d^6^4s^2^, while the configuration of Co^2+^ is 3d^5^4s^2^. The presence of a large number of unpaired highly localized electrons (electrons that are not participating in the formation of chemical bonding and conductivity) in Co^2+^ (3d^5^) allows the formation of a larger dipole moment during polarization in the high-frequency region. This is reflected in the larger amplitude of the local minimum on the frequency dependence of the real part of permittivity. With an increase in the Ni^2+^ concentration (Ni^2+^ ion with a smaller number of unpaired highly localized electrons – only 4 unpaired electrons per 3d^6^ orbitals), the contribution to polarization losses decreases.

**Fig. 7:**
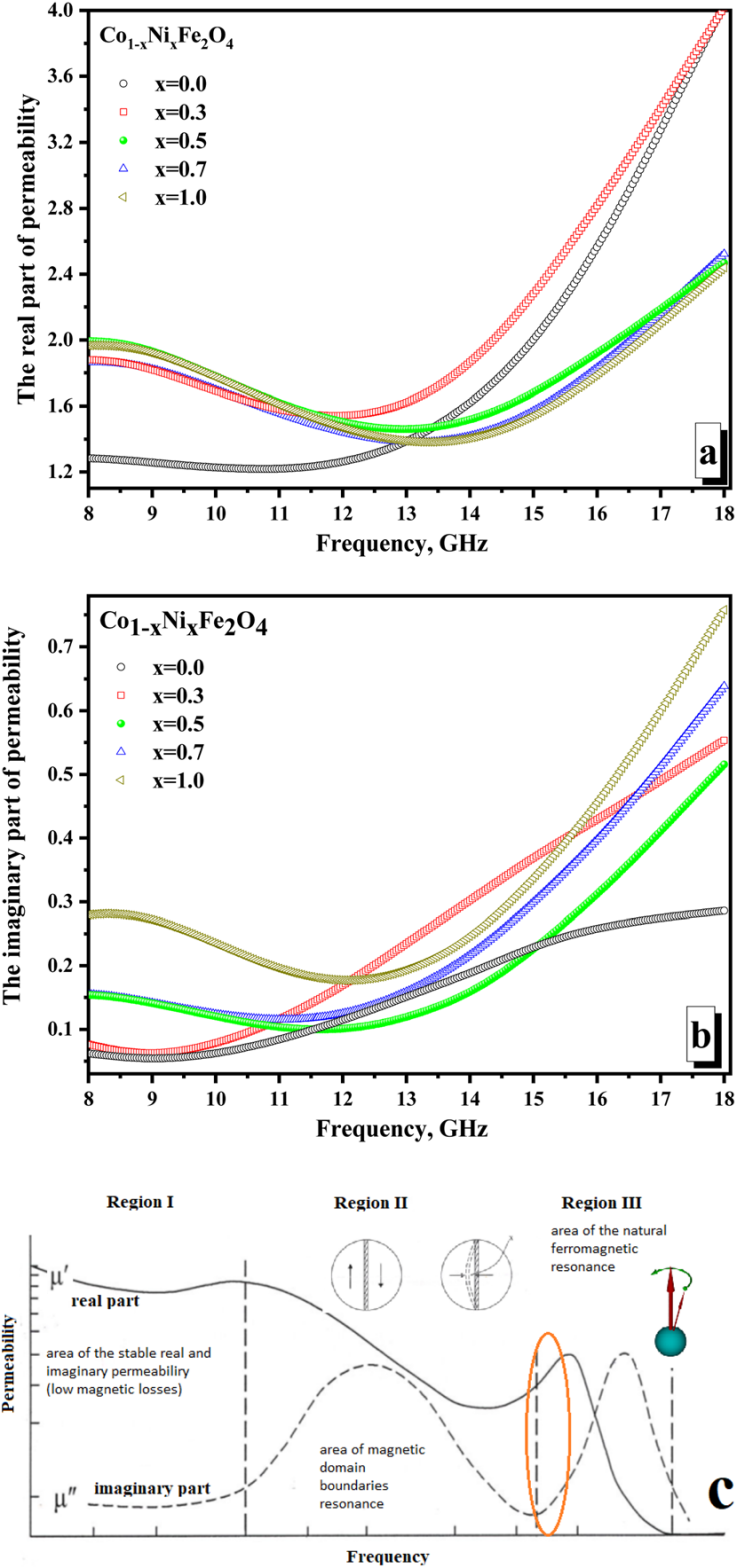
Frequency dependences of the permeability of CNFO (*x* = 0.0–1.0). (a) Real part of the permeability; (b) imaginary part of permeability; (c) mechanism of the magnetic losses in condensed matter in the high-frequency range.

Fig. 7 illustrates the frequency-dependent behavior of magnetic permeability. It is widely recognized that the magnetic permeability's real part arises from the reversal processes of magnetization. Simultaneously, the magnetic permeability's imaginary part is accountable for losing the resonant nature – the domain boundaries resonance (DBR) and the natural ferromagnetic resonance (NFMR). The first type of resonance (DBR) is associated with the intense absorption of electromagnetic radiation energy at frequencies corresponding to the internal frequencies of the domain wall's motion. DBR is noted at lower frequencies compared to the NFMR. The second type of resonance (NFMR) is caused by the intense absorption of electromagnetic energy at frequencies corresponding to the precession frequency of the magnetic moment of the electronic subsystem. When analyzing Fig. 7a, it can be noted that at frequencies up to 12 GHz, the minimum values (∼1.21–1.26) of the real part of the magnetic permeability are characterized by the *x* = 0 sample (pure cobalt ferrite).

All other samples have higher values (in the range of ∼1.45–1.99) of actual permeability. This can be revealed by the fact that cobalt ferrite has higher values of magnetocrystalline anisotropy and coercive force, which requires more energy for the magnetization reversal processes and makes it difficult for the electromagnetic flux to pass through the material (lower permeability values on frequency dependences). With an increase in the concentration of Ni^2+^ ions, the values of the magnetocrystalline anisotropy and coercive force decrease, which increases the values of the magnetic permeability and results in an increase in the real part of the magnetic permeability. At frequencies above 12 GHz, the situation changes. The maximum values (reaching 4.0) characterize samples with the maximum concentration of cobalt (*x* = 0.0 and 0.3). This may be due to the influence of the higher magnetic moment of the cobalt ion compared to the nickel ion. Relatively low values of the imaginary part of the permeability (Fig. 7b) and the absence of a clearly defined peak in the frequency dependences may indicate the absence of intense absorption due to magnetic losses of a resonant nature.

The minimum values of the imaginary part (∼0.06–0.28) are noted for the *x* = 0.0 sample (which can also be explained by the higher value of the magnetocrystalline anisotropy for pure cobalt ferrite). The maximum values of the imaginary part are noted (∼0.29–0.76) for the *x* = 1.0 sample. Let's analyze the frequency dependences of the real and imaginary parts of the magnetic permeability. We can conclude that the studied frequency range (8–18 GHz) is in the intermediate region between the two resonances. Fig. 7c shows the mechanism that explains the behavior of magnetic permeability with increasing frequency of electromagnetic radiation. A slight joint increase in both the real and imaginary parts of the magnetic permeability indicates the transition of the magnetic loss mechanism from domain wall resonance to natural ferromagnetic resonance. The energy losses in reflection can be expected to be a combination of electrical and magnetic losses due to polarization processes (dipole polarization) and magnetization reversal processes in the region of inter-resonant processes.

**Fig. 8:**
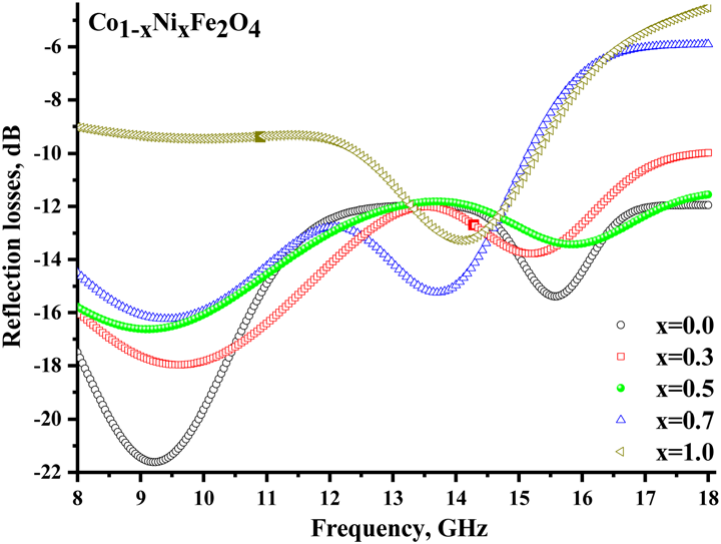
Frequency dependences of the reflection losses of CNFO (*x* = 0.0–1.0).

Fig. 8 illustrates the frequency-dependent behavior of the reflection coefficient (reflection losses), representing the reflected wave's energy losses. Negative values of the coefficient indicate the attenuation of the reflected radiation. It should be noted that for all samples (except *x* = 1.0), significant reflection losses were noted (with a reflection coefficient of more than −10 dB). For each sample (except *x* = 1.0), 2 broad peaks can be distinguished on the frequency dependences. Smaller peaks at frequencies above 13 GHz may be due to the combined energy losses due to magnetic and electrical losses. At the same time, peaks in the range of up to 13 GHz can result from multiple reflection processes in the material itself and processes of superposition of the reflected and incident waves.

## Conclusions

CNFO or Co_1−*x*_Ni_*x*_Fe_2_O_4_ (*x* = 0.0–1.0) nanoparticles have been produced using a chemical method (citrate-nitrate auto combustion technique). All samples have been examined using XRD, which has confirmed the formation of the cubic spinel ferrite structure (space group *Fd*3̄*m*) without any detectable impurities (single phase). The lattice parameter and unit cell volume behavior correlate well with the average ionic radii of Co^2+^ and Ni^2+^ ions and their coordination numbers. Thus, an increase in the Ni^2+^ content from *x* = 0.0 to *x* = 1.0 leads to a decrease in the lattice parameter (from 8.3805 to 8.3316 Å) and unit cell volume (from 58.86 to 57.83 Å^3^). The purity of the chemical composition (absence of undesirable impurities or phases) and the successful synthesis of nanosized spinel ferrites Co_1−*x*_Ni_*x*_Fe_2_O_4_ have been confirmed by the EDXS data. The obtained compositions of the Co_1−*x*_Ni_*x*_Fe_2_O_4_ system are homogeneous and contain small spherical particles with unevenly oriented grains in the form of aggregates, which was established during the microstructure analysis. Through the analysis of TEM images, the mean particle size was determined, and these values exhibited good agreement with the average crystallite size derived from XRD data using the Scherrer equation. Fourier Transform Infrared (FTIR) studies indicated the formation of hydrogen bonds amongst hydroxyl groups in Co_1−*x*_Ni_*x*_Fe_2_O_4_ spinel ferrites, suggesting the existence of either adsorbed or free water within the samples. The observed variation in the force constants of the tetrahedral and octahedral sites was elucidated through the cation redistribution that occurred between these positions, a process triggered by alterations in grain size. Furthermore, the characteristics of the microwave properties were examined based on the *S*-parameters measured within the frequency range of 8–18 GHz. After analyzing the frequency dispersions of the permittivity and permeability, the main mechanisms of the EMR interaction with condensed matter in the high-frequency range were discussed. It was assumed that the energy losses due to reflection are a combination of electrical and magnetic losses due to polarization processes (dipole polarization) and magnetization reversal processes in the region of inter-resonant processes. A significant attenuation of the reflected wave energy (−10 … −21.8 dB) opens broad prospects for practical applications. Finally, overall wave-absorbing materials are crucial for various applications, ranging from electromagnetic interference shielding to radar absorption. The prepared CNFO material offers several advantages over wave-absorbing materials, such as carbon materials, ceramics, conductive polymers, *etc.*, as follows: (1) tunability: the ability to adjust the Co to Ni ratio in the CNFO allows for the fine-tuning of the magnetic properties. This provides flexibility in tailoring the material's performance for specific frequency ranges or applications. (2) Enhanced crystallinity: as evident from the sharp and narrow XRD peaks, the CNFO material possesses high crystallinity. This characteristic often leads to improved electromagnetic properties and better stability. (3) High magnetic permeability: CNFO materials exhibit higher values of actual magnetic permeability, especially in the presence of increased Ni^2+^ ions. Higher magnetic permeability is beneficial for electromagnetic wave absorption as it facilitates the penetration of electromagnetic waves into the material, leading to increased absorption. (4) Balanced magnetic properties: the CNFO material balances the high magnetic moment of cobalt with the tunability provided by nickel. This balance ensures optimized absorption across a wide frequency range.

## Author contributions

Marwa M. Hussein – investigation (conducting a research and investigation process, specifically performing the experiments, or data/evidence collection); data curation (management activities to annotate (produce metadata)); Samia A. Saafan – investigation (conducting a research and investigation process, specifically performing the experiments, or data/evidence collection); data curation (management activities to annotate (produce metadata)). H. F. Abosheiasha – investigation (conducting a research and investigation process, specifically performing the experiments, or data/evidence collection); data curation (management activities to annotate (produce metadata)). Di Zhou – investigation (conducting a research and investigation process, specifically performing the experiments, or data/evidence collection); data curation (management activities to annotate (produce metadata)). D. S. Klygach – investigation (conducting a research and investigation process, specifically performing the experiments, or data/evidence collection); data curation (management activities to annotate (produce metadata)); formal analysis (application of statistical, mathematical, computational, or other formal techniques to analyze or synthesize study data). M. G. Vakhitov – data curation (management activities to annotate (produce metadata)); formal analysis (application of statistical, mathematical, computational, or other formal techniques to analyze or synthesize study data); S. V. Trukhanov – investigation (conducting a research and investigation process, specifically performing the experiments, or data/evidence collection); methodology (development or design of methodology; creation of models). A. V. Trukhanov – investigation (conducting a research and investigation process, specifically performing the experiments, or data/evidence collection); supervision (oversight and leadership responsibility for the research activity planning and execution, including mentorship external to the core team); conceptualization (ideas; formulation or evolution of overarching research goals and aims); funding acquisition (acquisition of the financial support for the project leading to this publication). T. I. Zubar – investigation (conducting a research and investigation process, specifically performing the experiments, or data/evidence collection); formal analysis (application of statistical, mathematical, computational, or other formal techniques to analyse or synthesize study data) and methodology (development or design of methodology; creation of models); K. A. Astapovich – investigation (conducting a research and investigation process, specifically performing the experiments, or data/evidence collection); formal analysis (application of statistical, mathematical, computational, or other formal techniques to analyse or synthesize study data); draft preparation. Hesham M. H. Zakaly – investigation (conducting a research and investigation process, specifically performing the experiments, or data/evidence collection); formal analysis (application of statistical, mathematical, computational, or other formal techniques to analyse or synthesize study data) and methodology (development or design of methodology; creation of models). Moustafa A. Darwish – investigation (conducting a research and investigation process, specifically performing the experiments, or data/evidence collection); supervision (oversight and leadership responsibility for the research activity planning and execution, including mentorship external to the core team); formal analysis (application of statistical, mathematical, computational, or other formal techniques to analyse or synthesize study data) and methodology (development or design of methodology; creation of models); funding acquisition (acquisition of the financial support for the project leading to this publication).

## Conflicts of interest

There are no conflicts to declare.

## Supplementary Material
